# Impact of a structured case report on self-reported responses to simulated emergency scenarios: a randomized survey-based study

**DOI:** 10.1038/s41598-026-54854-w

**Published:** 2026-05-25

**Authors:** Christian Porschen, Katharina E. M. Hellenthal, Alexander Zarbock, Michael Fujarski, Paul Brauckmann, Thi Ngoc-Anh Nguyen, Sebastian Rehberg, Jan Wnent, Matthias Lange

**Affiliations:** 1 Department of Anesthesiology, Intensive Care and Pain Medicine, University Hospital Muenster, Albert-Schweitzer-Campus 1, A1, 48149 Münster, Germany; 2 Division of Pulmonary and Critical Care Medicine, Massachusetts General Hospital, Harvard Medical School, Boston, MA USA; 3 Institute of Medical Informatics, University of Muenster, Muenster, Germany; 4 Institute for Translational Psychiatry, University of Muenster, Muenster, Germany; 5 Department of Anesthesiology, Intensive Care, Emergency Medicine, Transfusion Medicine and Pain Therapy, Medical School and University Medical Center OWL, Bielefeld University, Campus Bielefeld-Bethel, Bielefeld, Germany; 6 Institute for Emergency Medicine, University Hospital Schleswig-Holstein, Kiel, Germany; 7 Department of Anesthesiology and Intensive Care Medicine, University Hospital Schleswig-Holstein, Kiel, Germany

**Keywords:** Airway management, Trauma care, Case reports, Emergency decision-making, Prehospital medicine, Diseases, Health care, Medical research

## Abstract

Case reports are a long-standing component of medical research, but their association with self-reported responses to simulated clinical scenarios remains underexplored. This study investigated whether exposure to a structured case report influences simulated emergency medical decisions. This randomized survey-based study was conducted among licensed physicians between March and May 2025. Participants were randomly assigned to an intervention group, which received a structured case report on blunt neck trauma, or a control group, which did not. Both groups subsequently provided self-reported Likert-scale responses to identical simulated emergency scenarios, covering five domains: local trauma consequences, accompanying injuries, diagnostic methods, airway management strategies, and hospital selection. Responses were collected on Likert scales. Group differences were assessed using Mann–Whitney U tests, t-tests, and chi-squared tests; prespecified exploratory subgroup analyses examined the moderating role of clinical experience. Sixty-three participants completed the study. Baseline demographics and qualifications did not differ significantly between groups. Exposure to the case report was associated with statistically significant differences in self-reported responses to simulated scenarios, with the most pronounced associations in airway management strategies (all five items statistically significant, median effect size r = 0.44). Intervention participants assigned higher ratings to maintaining spontaneous breathing and supraglottic airway use, while assigning lower appropriateness to endotracheal intubation and cricothyrotomy. Moderate associations were observed in diagnostic choices (e.g., pathological breath sounds, ultrasound use) and hospital selection (e.g., ECMO availability). No consistent effects were found for local or accompanying injuries. Exploratory stratified analyses revealed that junior physicians (≤ 5 years of practice) appeared more responsive, with significant differences across multiple domains and larger effect sizes (median r > 0.6). In contrast, no statistically significant between-group differences were detected in the senior (> 15 years) subgroup, although this exploratory analysis was limited by small subgroup size. Structured case reports may influence self-reported responses to simulated emergency scenarios, particularly among less experienced clinicians. These findings suggest that the educational impact of case-based learning formats may vary depending on the level of clinical experience, but do not allow conclusions about real-world clinical behavior or patient outcomes. Future work should further investigate adaptive case report formats and their potential integration into emergency medicine training.

*Trial registration*: German Clinical Trials Register (DRKS00038961), registered von 13 January 2026.

## Introduction

Clinical case reports have a long-standing tradition as key components of medical literature and training. Since the early days of scientific medicine, they have provided a foundation for identifying research questions, and they continue to play a crucial role in generating hypotheses for studies, including randomized controlled trials^1^. In 2007, the first PubMed-indexed journal exclusively devoted to case reports—*Journal of Medical Case Reports*—was launched. Many other journals remain dedicated sections for case reports, for example *The Case Records of Massachusetts General Hospital* in the *New England Journal of Medicine* or the *Clinical Case Conference* in the *American Journal of Psychiatry*.

Case reports may present rare or unusual observations, accumulate scientific data on uncommon disorders, or help generate new hypotheses^2^. They also represent an important tool in pharmacovigilance^3,4^. However, they have limitations, including the absence of epidemiological parameters, the inability to establish causal relationships, and the risk of overinterpretation^5,6^.Citation numbering is shifted here compared with the submitted manuscript version.

Clinical medical education is often based on case reports. Typical case vignettes are regularly included in textbooks and lectures, serving as catalysts in diagnostic reasoning, management strategies, and clinical decision-making^1,7,8^. Despite their widespread use as an educational resource, little is known about how case reports influence medical decision-making at different stages of training.

In emergency medicine, where structured scientific investigations are often difficult to conduct and therefore limited in number, case reports may be particularly valuable as an educational tool^6,9^. In the present survey-based trial, we sought to determine whether reading a structured case report is associated with differences in simulated emergency medical decision-making.

## Materials and methods

### Study design and participants

This randomized survey-based study employed a meta-research design to examine whether reading a clinical case report in emergency medicine is associated with differences in self-reported responses in simulated clinical scenarios. Data were collected via an anonymous online survey distributed to practicing licensed physicians. Participants were recruited using a convenience and snowball sampling approach. Initial invitations were sent via email to various German emergency medical services, anesthesiology, and emergency departments, as well as to the mailing list of an emergency medical working group between March and May 2025. Participants were encouraged to share the survey with colleagues, allowing for indirect recruitment across a broader clinical network. No additional inclusion or exclusion criteria were applied.

Before participating in the survey, all participants received a written data protection and consent form that clearly explained the purpose of the study, the type of data collected, the storage period, and the participant´s rights under the European general data protection regulation (GDPR). Participation was voluntary and only possible after active consent was obtained before the survey began. The study was approved by the Ethics Committee of the Medical Association of Wesphalia-Lippe and the University of Münster, Germany (approval ID: 2025-191-f-S). The study was conducted in accordance with Declaration of Helsinki. This study was retrospectively registered in the German Trial Register (DRKS), a primary registry participating in the WHO International Clinical Trial Registry Platform (ICTRP) (DRKS-ID: DRKS00038961). Registration was completed on 13 January 2026.

### Randomization and intervention

Upon accessing the survey platform, participants were randomly assigned using computer-generated randomization to one of two groups. Group 1 (intervention group) received a structured case report on blunt neck trauma that had been accepted for publication in a peer-reviewed journal, but was unpublished at the time of the study^10^. After reading the report, participants were presented with brief clinical scenarios and answered a standardized set of questions regarding their confidence in clinical decisions. Group 2 (control group) received only the brief clinical scenarios without prior exposure to the case report and answered the same set of questions. The no-exposure control condition was selected as a pragmatic initial comparator to assess immediate differences associated with case-report exposure. This design was not intended to distinguish the specific effect of a case report from the broader effect of reading any educational material. Participants could not be blinded to allocation because the intervention required visible exposure to the case report before the scenario questions. Both groups nevertheless received the same scenario questions, and responses were collected anonymously.

### Case report

The case report was presented exclusively to participants in group 1. It describes a 16-year-old motorcyclist who sustained a complete laryngotracheal separation (Fuhrmann grade V) after colliding with a wire fence. The patient presented with stridor, aphonia, and progressive subcutaneous emphysema. Initial intubation attempts failed despite adequate visualization and successful passage of the tube through the glottis during laryngoscopy. Adequate ventilation was subsequently achieved using a supraglottic airway (laryngeal mask). During helicopter transport, the patient developed bilateral subcutaneous emphysema, prompting needle decompression. At the trauma center, emergency surgical exploration revealed complete tracheal separation at the third tracheal ring with a 3 cm gap, necessitating immediate surgical tracheostomy and anastomosis. The patient recovered well, but with bilateral recurrent nerve palsy and was discharged after 14 days with a tracheostomy tube^10^.

### Clinical scenarios

Two brief simulated scenarios closely aligned with key elements of the case report were presented to both groups, accompanied by the same set of questions. This deliberate overlap was intended to assess immediate self-reported responses after exposure to the case report, not transfer to unrelated clinical scenarios. Responses were recorded using a 5-point Likert scale (1 = very unlikely/not useful to 5 = very likely/very useful) across five domains:

- Suspected local trauma consequences: expected injuries including laryngeal edema, recurrent nerve palsy, neck hematoma, laryngeal fractures, and tracheal rupture.
- Suspected accompanying injuries: concomitant injuries such as traumatic brain injury, pulmonary contusions, pneumothorax, spinal fractures, and vascular dissections.
- Diagnostic methods: utility of palpation, auscultation, dyspnea assessment, pathological breath sounds, and ultrasound examination.
- Airway strategies: appropriateness of maintaining spontaneous breathing, controlled mask ventilation, use of supraglottic airway devices, endotracheal intubation, and emergency cricothyrotomy.
- Hospital selection criteria: importance of transport distance, bronchoscopy availability, trauma center capabilities, emergency surgical airway options, and ECMO availability.
- The primary analysis compared response distributions between the intervention and control groups across the predefined Likert-scale scenario items. Domain-level summaries and all experience-stratified analyses were exploratory and hypothesis-generating.

### Participant characteristics

Demographic and professional experience data were collected, including age, gender, years of medical practice, years in emergency services, and professional qualifications (specialist status, board certification in emergency medicine, and board certification in intensive care). Participants were stratified into three groups based on years of medical practice: junior physicians (≤ 5 years of medical practice), experienced physicians (6–15 years of medical practice), and senior physicians (≥ 15 years of medical practice).

### Patient and public involvement

Patients or members of the public were not involved in the design, conduct, reporting, or dissemination plans of this study. The study focused exclusively on emergency physicians, and no patient or public input was obtained.

### Statistical analysis

Primary analysis compared groups using Mann–Whitney U tests for ordinal Likert scale data, with independent t-tests as sensitivity analyses. For categorical variables, Chi-squared test was used as appropriate, depending on data distribution. Effect sizes were calculated using r for Mann–Whitney U tests and Cohen’s d for t-tests. Effect size interpretation followed Cohen’s conventions: small (r ≥ 0.1, d ≥ 0.2), medium (r ≥ 0.3, d ≥ 0.5), and large (r ≥ 0.5, d ≥ 0.8). Normality was assessed using Shapiro–Wilk tests, Bonferroni correction was applied when testing multiple variables within domains.

Exploratory subgroup analyses were performed stratified by experience level. Interaction effects between group assignment and experience were tested using Kruskal–Wallis tests. Multiple comparison correction was applied using the Bonferroni method. Statistical significance was set at *p* < 0.05. As an adjusted sensitivity analysis, one ordinal logistic regression model was fitted for each Likert-scale item. Each model included case-report exposure as the main exposure and adjusted for years of medical practice, gender, emergency medicine certification, and intensive care certification. Adjusted models used complete-case analysis per outcome item. All analyses were performed using Python 3.12 with scipy.stats, pandas, and statsmodels libraries.

## Results

### Participant characteristics

A total of 63 physicians completed the study (Group 1: n=30, Group 2: n=33). Mean age was not significantly different between groups. Gender distribution showed 80.0% male participants in Group 1 and 66.7% in Group 2 (*p*=0.365). Years of medical practice ranged from 1–37 years (mean: 9.3 years), with no significant difference between groups (*p*=0.193). Years in emergency services ranged from 0–35 years (mean: 7.2 years), also without significant group differences (*p*=0.645).

Professional qualifications were balanced between groups: specialist certification (Group 1: 46.7%, Group 2: 54.5%), emergency medicine certification (Group 1: 33.3%, Group 2: 48.5%), and intensive care certification (Group 1: 20.0%, Group 2: 45.5%), with none of the above reaching cut-off significance levels. An overview of the participant characteristics is demonstrated in Table 1.

**Table 1:** Baseline characteristics by group assignment. Data presented as n (%) for categorical variables and mean (SD) for continuous variables. Statistical tests: χ^2^ for categorical variables, Mann–Whitney U, or t-test for continuous variables.

Characteristics	Group 1 (Case Report)	Group 2 (Control)	Total	p-value
Male gender, n (%)	24 (80.0)	22 (66.7)	46 (73.0)	0.365
Years medical practice, mean (SD)	9.3 (8.7)	12.2 (10.1)	10.9 (9.5)	0.193
Years emergency services, mean (SD)	7.2 (8.7)	7.4 (10.2)	7.3 (9.5)	0.645
Specialist certification, n (%)	14 (46.7)	18 (54.5)	32 (50.8)	0.710
Emergency service expertise, n (%)	10 (33.3)	16 (48.5)	26 (41.3)	0.335
Emergency medicine certification, n (%)	19 (63.3)	17 (51.5)	36 (57.1)	0.489
Intensive care certification, n (%)	6 (20.0)	15 (45.5)	21 (33.3)	0.061

### Primary outcomes

A series of Mann–Whitney U tests was conducted to assess differences between groups across five predefined variable categories: suspected local trauma consequences, suspected accompanying injuries, diagnostic methods, airway strategies, and hospital selection. Both statistical significance (*p*-values) and effect sizes (r) were reported to quantify the magnitude of between-group differences.

The category “airway strategies” yielded the most pronounced group differences, with the majority of comparisons showing significant *p*-values (*p* < 0.001) and large effect sizes (median r > 0.4), indicating larger between group differences within this sample. Specifically, participants exposed to the case report rated maintenance of spontaneous breathing significantly higher (*p* = 0.005, r = 0.361) and showed increased preference for supraglottic airway devices (*p* = 0.001, r = 0.622). Conversely, the case report group rated both endotracheal intubation (*p* = 0.002, r = 0.404) and emergency cricothyrotomy as less appropriate in the simulated scenario (*p* = 0.013, r = 0.324).

The category “diagnostic methods” demonstrated moderate effect sizes (r ~ 0.25), with several comparisons reaching statistical significance ( *p*< 0.05), suggesting detectable but less pronounced, differences between groups. In detail, the case report group placed greater emphasis on assessment of pathological breath sounds (*p* = 0.026, r = 0.287), while rating ultrasound examination as less useful (*p* = 0.012, r = 0.324).

In the category “hospital selection” the results were heterogeneous. While some comparisons showed statistically significant p-values, the overall effect size ranged from small to moderate (r ~ 0.1–0.4), indicating variability in the observed associations across items. Notably, the case report group placed significantly higher importance on ECMO availability (*p* = 0.001, r = 0.429).

The categories “accompanying injuries” and “local trauma consequences” yielded non-significant *p*-values (*p* > 0.05) in most comparisons, with small or negligible effect sizes (r < 0.2). Participants who read the case report showed statistically significant differences in the assessment of specific potential complications, rating tracheal rupture (*p* = 0.029, r = 0.276) and pneumothorax (*p* = 0.011, r = 0.322) as more likely.

**Fig. 1:**
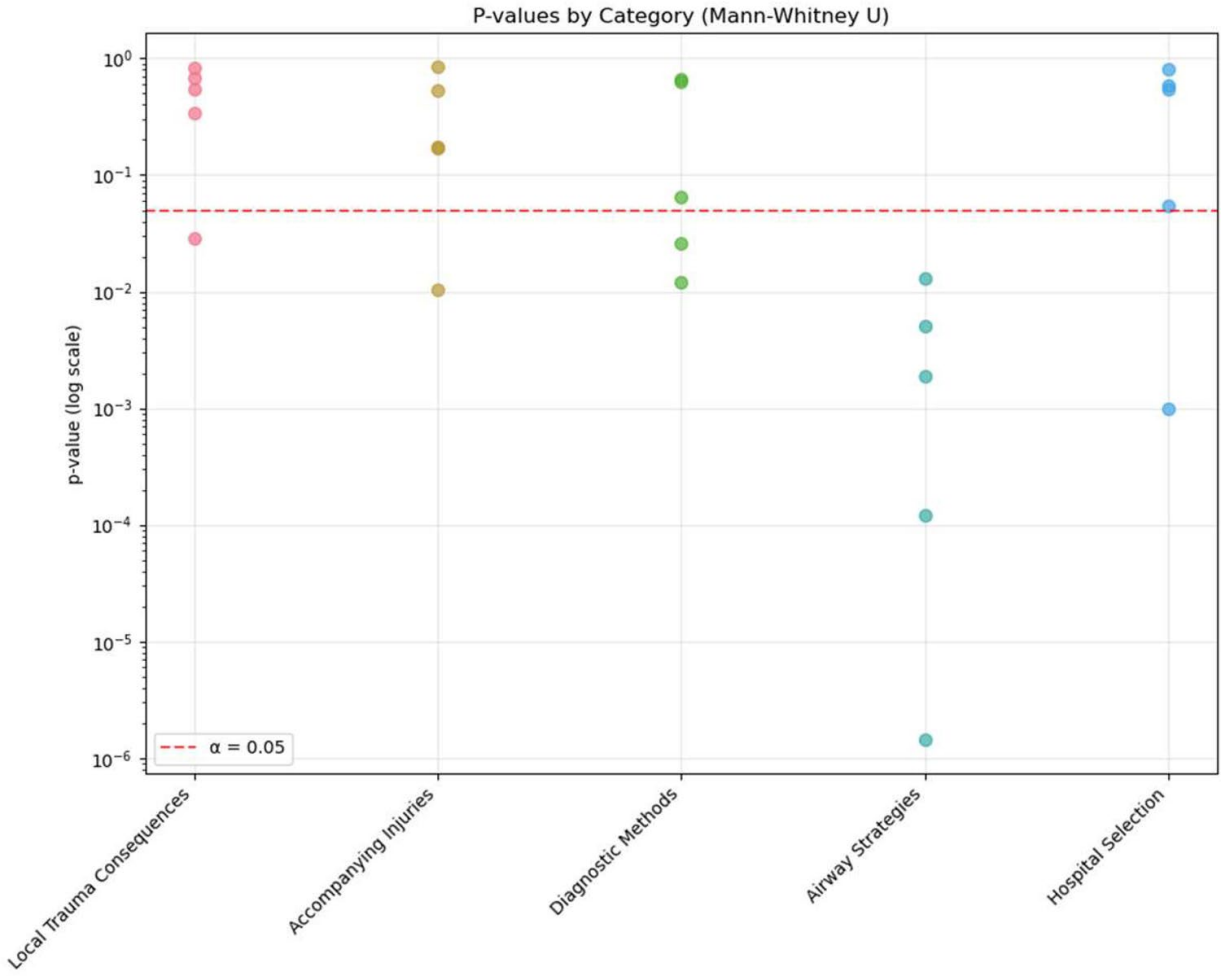
Distribution of *p*-values by clinical domain from Mann–Whitney U tests comparing case report and control groups. *P*-values from statistical comparisons between groups are displayed on a logarithmic scale, with each point representing one tested variable within the respective clinical domain. The red dashed line indicates the significance threshold (α = 0.05). Points below this line represent statistically significant differences between groups. Airway Strategies contained the highest number of statistically significant comparisons, including several with *p*-values < 0.001, whereas other domains showed fewer significant comparisons. Within this sample, statistically significant between-group differences were more frequently observed for airway-related scenario items than for other domains.

**Fig. 2:**
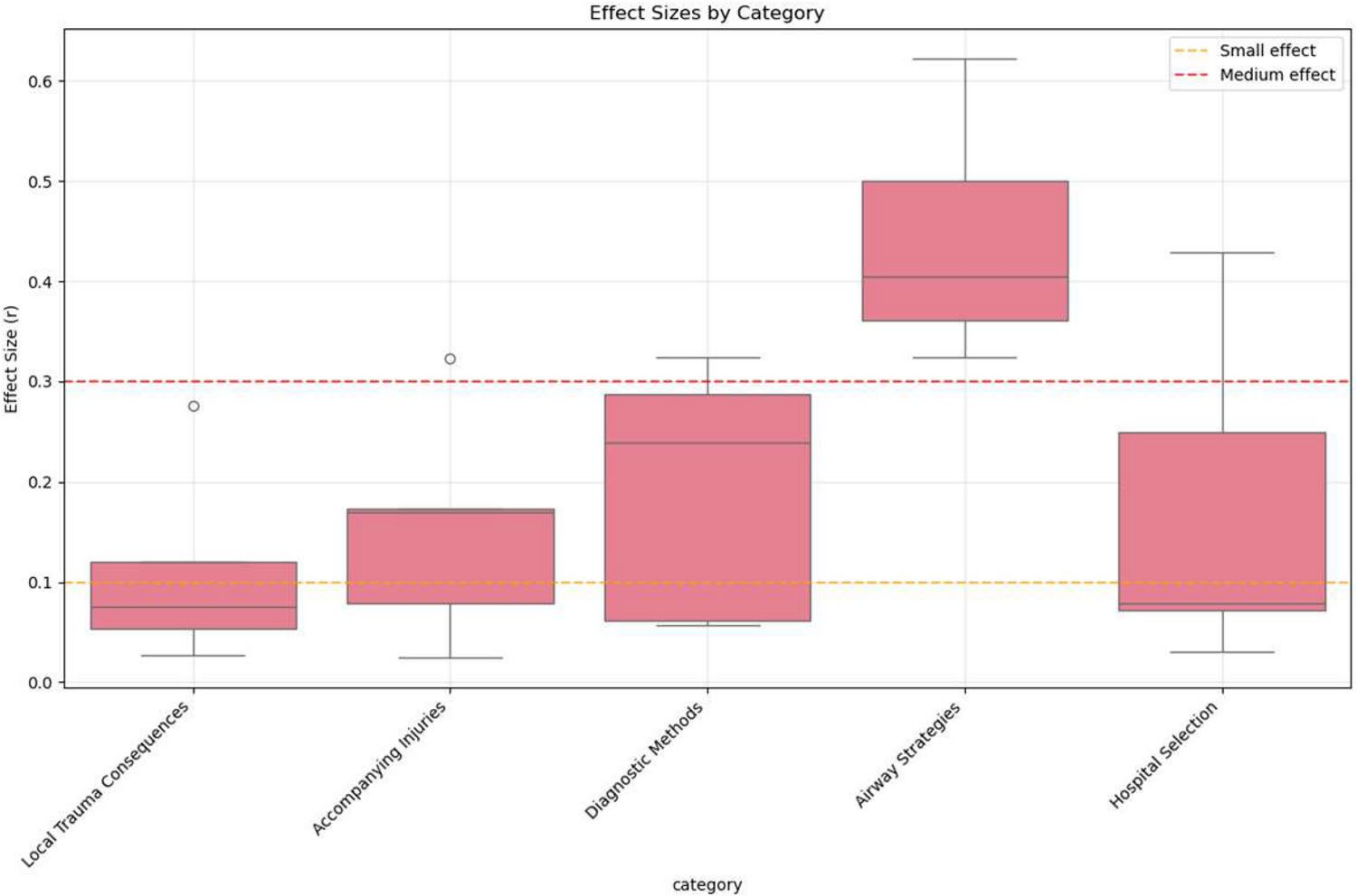
Effect sizes (r) by clinical domain for all tested variables. Box plots display the distribution of effect sizes from Mann–Whitney U tests across clinical domains. The orange dashed line indicates small effects (r = 0.1) and the red dashed line indicates medium effects (r = 0.3). Airway Strategies showed comparatively larger effect-size estimates than the other domains, whereas Local Trauma Consequences and Accompanying Injuries generally showed smaller effect sizes. Hospital selection and Diagnostic Methods displayed moderate variability across items. Within this sample, larger effect-size estimates were primarily observed for airway-related self-reported decision items.

A summary of statistical results is presented in Table 2 as well as Figs. 1 and 2, showing the −log_10_-transformed p-values and corresponding effect sizes for each comparison across the five categories.

**Table 2:** Statistical test results for confidence in self reported decision-making variables. Results of Mann–Whitney U tests comparing responses between case report group (Group 1) and control group (Group 2) across all clinical domains. Data presented as mean (SD) for each group. Effect sizes (r) calculated using the standard formula for Mann–Whitney U tests. Statistical significance: *p* < 0.05. Effect size interpretation follows Cohen’s conventions: small (0.1–0.3), medium (0.3–0.5), and large (≥ 0.5). Airway Strategies demonstrated the most consistent and largest effects, with all variables showing statistical significance and effect sizes ranging from small to large. These estimates should be interpreted cautiously because responses were ordinal and the sample was small. Abbreviations: SD, standard deviation.

Domain	Total tests	Significant differences (%)	Mean effect size	Large effects (r ≥ 0.5)	Medium effects (0.3 ≤ r < 0.5)	Small effects (0.1 ≤ r < 0.3)
Local trauma consequences	5	1/5	0.110	0	0	2
Accompanying injuries	5	1/5	0.154	0	1	2
Diagnostic methods	5	2/5	0.194	0	1	2
Airway strategies	5	5/5	0.442	2	3	0
Hospital selection	5	1/5	0.172	0	1	1

### Outcomes by clinical experience

To investigate whether clinical experience may moderate the effect of case report exposure on simulated decision-making, participants were exploratory stratified into three levels based on years of professional experience: junior (≤ 5 years), experienced (6–15 years), and senior (≥ 15 years). For each group, the percentage of statistically significant changes and effect size in their confidence in clinical decisions associated with exposure to the case report were calculated.

The proportion of statistically significant changes in decision-making tended to decrease with increasing experience, and effect sizes (r) of significant changes were generally larger in less experienced physicians.

**Fig. 3:**
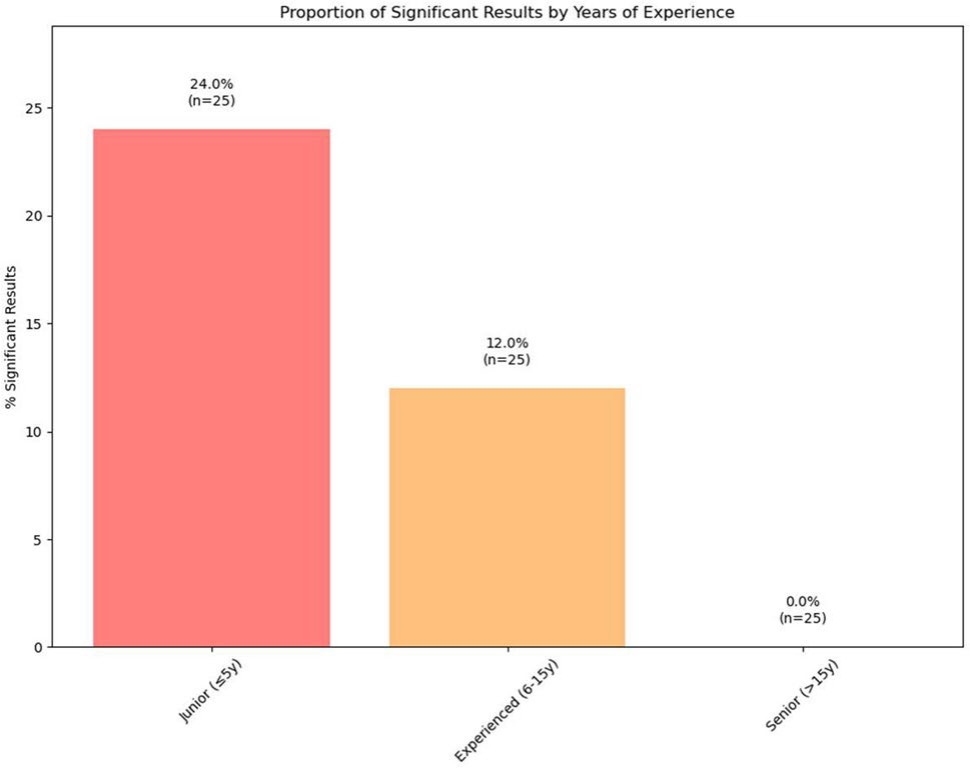
Proportion of significant results by years of medical experience. Percentage of statistical tests achieving significance (*p* < 0.05) within each experience stratum, with sample sizes indicated. In this exploratory subgroup analysis, a higher proportion of statistically significant between-group comparisons was observed among junior physicians (≤ 5 years) than among more experienced physicians. No statistically significant comparisons were observed in the senior subgroup (> 15 years), although subgroup sample sizes were limited.

**Fig. 4:**
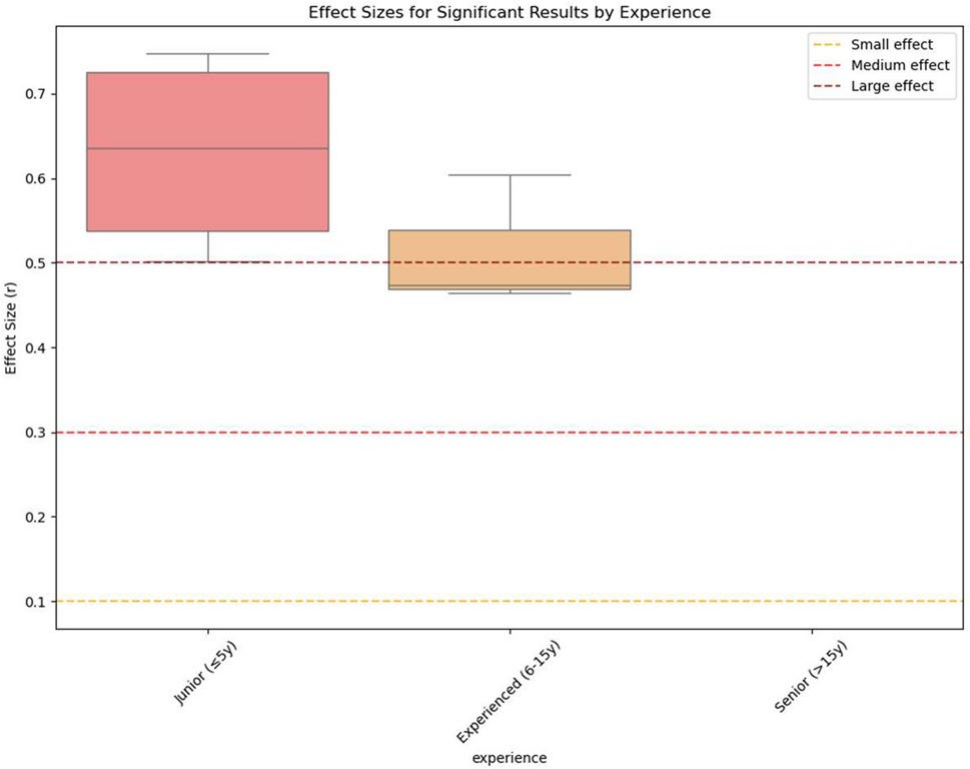
Effect sizes for significant results by experience level. Distribution of effect sizes (r) for statistically significant comparisons only, stratified by years of medical experience. Reference lines indicate small (r = 0.1), medium (r = 0.3), and large (r = 0.5) effect thresholds. Among statistically significant comparisons, lager effect-size estimates were observed in the junior physician subgroup (≤ 5 years) than in the experienced subgroup (6–15 years). No statistically significant comparisons were identified in the senior subgroup; however, subgroup sizes were small.

Junior physicians showed significant effects across all assessed clinical domains, with airway strategies being most affected (4/5 items significant). Experienced physicians (6–15 years) demonstrated a lower degree of differences after case report exposure, with 3 out of 25 comparisons reaching statistical significance (12%). While fewer domains were affected compared to junior physicians, effect sizes remained large (mean r = 0.51). The effects were primarily concentrated on airway strategies and diagnostic methods. Physicians with more than 15 years of experience showed no statistically significant associations with case report exposure, with none of the 25 comparisons reaching statistical significance. Table 3 summarizes these results. Formal interaction testing revealed significant group × experience interactions for 12 variables (*p* < 0.05), confirming that the magnitude of case report influence systematically varies with clinical experience. Mean significance levels and effect sizes by category and experience are depicted in Figs. 3 and 4, respectively.

**Table 3:** Experience-stratified analysis summary. Summary of case report influence stratified by years of medical practice. Sample sizes show total participants per experience level with Group 1 (case report) and Group 2 (control) distribution. One participant reported unrealistic constellations of years of experience and was therefore excluded from this analysis. Effect sizes calculated using Mann–Whitney U tests.

Experience level	Sample size	Group 1/Group 2	Significant results (%)	Mean effect size (All)	Mean effect size (Significant)	Primary affected domains
Junior (≤ 5 years)	27	15/12	6 (24.0)	0.269	0.630	Airway Strategies, Accompanying Injuries
Experienced (6–15 years)	20	8/12	3 (12.0)	0.257	0.514	Airway Strategies, Diagnostic Methods
Senior (> 15 years)	15	6/9	0 (0.0)	N/A	N/A	None

In the adjusted ordinal logistic regression sensitivity analysis, all 25 outcome models were estimable. After adjustment for years of medical practice, gender, emergency medicine certification, and intensive care certification, the direction of the association between case-report exposure and responses was consistent with the primary nonparametric analysis. The adjusted associations remained most pronounced for airway strategies: case-report exposure was associated with higher odds of rating maintenance of spontaneous breathing (adjusted odds ratio [aOR] 6.27, 95% CI 1.96 to 20.06), controlled mask ventilation (aOR 9.68, 95% CI 2.88 to 32.57), and supraglottic airway use (aOR 19.41, 95% CI 5.43 to 69.44) as more appropriate, and with lower odds of rating endotracheal intubation (aOR 0.18, 95% CI 0.06 to 0.55) and emergency cricothyrotomy (aOR 0.18, 95% CI 0.06 to 0.56) as more appropriate. The adjusted findings should be interpreted as exploratory sensitivity analyses rather than confirmatory evidence.

## Discussion

Case reports play a pivotal role in ongoing medical education and research, yet their impact on participants’ confidence in clinical decision-making in emergency scenarios remains underexplored^5,11^. In this randomized, survey-based study, we observed that exposure to a structured, peer-reviewed case report was associated with significant differences in simulated emergency medical decision-making. Notably, junior physicians showed greater responsiveness to case report exposure, whereas simulated decision-making of physicians with more than 15 years of clinical experience did not differ detectably between groups. Among junior physicians, this association was particularly pronounced in airway management decisions with statistically significant differences across all assessed airway-related items in the simulated setting.

The variability in how physicians at different experience levels interpret and apply information from case reports highlights the importance of tailoring educational materials to the learners´ stage.^9^ Junior physicians may benefit from detailed, narrative case reports that support step-by-step clinical reasoning, while experienced physicians might require more concise, guideline-focused content to integrate new insights efficiently. These considerations suggest that adaptive or experience-specific case report formats could enhance educational relevance, though this hypothesis requires further empirical evaluation^12,13^.

The findings of the present study suggest that both clinical experience and exposure to case-based educational material may be associated with differences in simulated decision-making in time-critical emergency situations, such as airway management. While junior physicians’ choices were more strongly associated with case report exposure, experienced physicians may have contextualized the presented information within broader clinical experience and existing guidelines. Although case reports represent a low level of evidence, their potential educational influence in emergency medicine warrants further consideration^11,14^. To maintain high standards in emergency education and training, medical case reports may benefit from a structured, well-compiled format and convey a clearly articulated narrative^6,7,12^^.^

Although the present study was designed to evaluate the effects of reading a case report on decision-making, it also includes exploratory secondary analyses that suggest that the extent of clinical experience and professional qualifications may moderate the association between case report exposure and decision-making outcomes. This observation could be interpreted in the light of the cognitive load theory and the resulting expertise reversal effect, which have previously been described in detail^15–17^. However, these exploratory findings should be interpreted cautiously, as the study was not powered to formally test mechanistic hypotheses and does not allow causal inference regarding experience-related effects. Nonetheless, the results may be considered in the context of ongoing discussions about qualification standards for physicians working in prehospital emergency care, as advocated by national medical societies^18,19^. Furthermore, telemedicine has been proposed as a potential means to offer advisory support by experienced physicians, thereby possibly improving potentially life-threatening medical conditions in emergency situations^20^. The adjusted ordinal regression analyses were added to address potential confounding but remain exploratory because the sample size was limited, several ordinal response categories were sparse, and complete-case modeling reduced the available sample for some items.

It can further be hypothesized, that case-based learning and confidence clinical decision-making may vary according to the physicians´ specialty background. For instance, surgeons may tend to focus on operative technical details—such as anatomical reconstruction and hemorrhage control—whereas anesthesiologists may prioritize airway management, ventilation strategies, and maintaining physiological stability during critical phases of illness. These differing clinical priorities could contribute to distinct learning outcomes and interpretations of the same case report. Future studies should therefore explore whether tailoring case report content to discipline-specific needs enhances its educational effectiveness.

It must be emphasized that the observations of the present study reflect only short-term effects on simulated decision-making. Whether the effects and knowledge gained from reading case reports in emergency medicine persist over time remains unknown. The study assessed multiple scenario items across several domains. Even with correction for multiple testing, individual statistically significant findings should be interpreted cautiously and require confirmation in larger, prospectively powered studies. Effect-size estimates from small samples of ordinal Likert responses should not be interpreted as direct evidence of educational importance or clinical relevance. Because experience strata were small, absence of statistical significance in the senior subgroup should not be interpreted as evidence of absence of association. Future studies with longer follow-up periods are needed to assess the durability and clinical relevance of these effects and to determine whether repeated exposure to case reports, integration into structured training programs, or reinforcement through clinical practice can enhance long-term retention and transfer to real-world practice. Furthermore, the interpretation of the results is constrained by the relatively small sample size, which may limit the external validity and generalizability of the findings. Moreover, due to the anonymous, open survey design, no reliable response rate can be determined, as the overall number of individuals who received or had access to the survey remains unknown. Recruitment by convenience and snowball sampling may have introduced selection bias. Physicians reached through professional networks or mailing lists may be more motivated, academically engaged, or familiar with continuous professional education than the broader emergency physician population. This may affect baseline knowledge, response patterns, and responsiveness to the intervention, limiting generalizability. A further limitation of the present study is that it focused exclusively on a single, specific emergency scenario involving catastrophic airway injury. While this allowed for a focused and controlled assessment of case report influence in that context, it remains unclear whether similar associations would be observed across other clinical contexts or emergency scenarios. Different emergency situations may present distinct challenges, cognitive demands, and decision-making processes, potentially leading to varying degrees of responsiveness to case report-based learning. Because the control group received no alternative educational material and there has been overlap between the key clinical scenario and the case report, the observed between-group differences may partly reflect a general studying, attention, or priming effect rather than the unique educational contribution of a case report. Future studies should include active comparators such as guideline excerpts, textbook passages, or other case-based formats. The lack of participant blinding and reliance on self-reported Likert-scale responses create a risk of performance and response bias. Participants exposed to the case report may have selected answers they perceived as consistent with the report rather than answers reflecting usual clinical reasoning or behavior. Future research should also explore a wider spectrum of emergency scenarios to better understand the transferability of our findings and to identify context-specific implications for the use of case reports in medical education. As a general limitation inherent to survey-based research, the artificial nature of simulated decision-making environments should be considered.

In conclusion, the present randomized survey-based study suggests that exposure to a structured medical case report is associated with differences in simulated decision-making among less experienced emergency physicians. However, identifying the underlying mechanisms by which emergency medicine case reports may contribute to learning and decision-making will be essential for the development, definition, and systematic evaluation of quality standards for future case report publications^6,7,12^.

## Data Availability

The datasets generated during and/or analyzed during the current study are not publicly available due to German data protection regulations but are available from the corresponding author on reasonable request.
